# Eggplant (*Solanum melongena* L.) polyphenol oxidase multi-gene family: a phylogenetic evaluation

**DOI:** 10.1007/s13205-014-0195-z

**Published:** 2014-01-29

**Authors:** Aravind Kumar Jukanti, Ramakrishna Bhatt

**Affiliations:** Central Arid Zone Research Institute, Jodhpur, 342003 Rajasthan India

**Keywords:** Eggplant, Polyphenol oxidase, Phylogenetic analysis, Multi-gene family, N-glycosylation

## Abstract

Polyphenol oxidases (PPOs) in different *Solanum* species including eggplant have been studied. PPOs have been implicated in undesirable enzymatic browning of eggplant fruit and also in plant defense. The main objective of this study was to identify and accelerate the further functional characterization of additional eggplant PPOs that are involved in food biochemistry and defense-related functions. Eggplant PPOs identified earlier were used in “Basic local alignment search tool (BLAST)” search against expressed sequence tag and nucleotide databases. We have identified seven additional sequences which were almost complete in length. The sequences of the PPOs were aligned and their phylogenetic and evolutionary relationships established. The sequences are quite diverse, broadly falling into two major clusters; three PPOs form a separate branch/minor cluster. The thirteen sequences had conserved copper A binding sites but copper B binding sites differed considerably in two new PPO sequences (AFJ79642 and ACR61398). A third conserved ‘Histidine-rich’ region has been identified at the ‘C’ terminus of the eggplant PPOs. In addition, all the seven new PPOs exhibited at least one glycosylated sequon in the mature PPO sequence. Identification of additional PPO genes will further help in functional and biological characterization of these PPOs.

## Introduction

Polyphenol oxidases (PPOs) can oxidize specific phenolic substrates in the presence of oxygen in contrast to peroxidases which oxidize phenols in presence of H_2_O_2_. PPOs are ubiquitously distributed in plants (Mayer and Harel 1979); they play a role in food quality and in plant defense against pest and pathogens (Thipyapong et al.^1995^; Thipyapong and Steffens 1997; Wang and Constabel 2004). PPOs are also involved in: time-dependent darkening and discoloration of cereal-based products ( Baik et al. 1994), biosynthesis of flavonoids (Ono et al. 2006) and in oxidation of flavonoids (Pourcel et al. 2005). PPOs come into contact with phenolic substrates that are released due to tissue damage. The phenols are oxidized to highly reactive *o*-quinones which either self-polymerize or further react with nucleophiles to produce dark colored pigments that are usually undesirable in fresh or processed foods (Anderson and Morris 2001).

PPOs contain two copper (Cu) binding sites (Cu A and Cu B) and the Cu ion is bound by conserved histidine residues. PPOs interact with molecular oxygen and phenolic substrates at Cu-A and Cu-B sites (Van Gelder et al. 1997). PPOs are nuclear encoded enzymes, synthesized as precursor proteins in cytosol, processed to mature proteins and imported into plastidial thylakoid membranes (Koussevitzky et al. 1998; Sommer et al. 1994). The typical N-terminal transit peptide of PPOs is about 80–100 amino acids in length and during the chloroplast import the molecular weight of the enzyme is reduced from ~65–70 to <60 kDa (Dry and Robinson 1994; Van Gelder et al. 1997). PPOs are basically three types based on the substrates they catalyze: cresolases (monophenol oxidases), *o*-diphenol oxidases (catecholases) and laccase-like multi-copper oxidases (Shetty et al. 2011). Among the three classes of PPOs, *o*-diphenol oxidases have been extensively characterized in plants. Catecholases mostly occur as multi-gene families, introns are absent among the dicotyledonous PPOs genes reported so far (Shetty et al. 2011). But introns have been reported in monocot species like pineapple, banana and wheat (Massa et al. 2007). Up-regulation of PPO genes upon mechanical wounding and damage due to pests has been reported in several crop species (Thipyapong et al. 1995, 1997; Wang and Constabel 2004).

The role of PPOs in plant defense and enzymatic discolouration/browning affecting quality of crop/plant products has led to extensive identification and characterization of PPO genes in several plant species. Identification and characterization of all/most PPOs in a plant species will aid in better understanding the structural and functional differences among the multi-gene family. Solanaceae crops like potato, tomato and eggplant/brinjal form an important part of the daily diet in many parts of the world. Specifically, eggplant is an important constituent of the Indian cuisine. The role of PPOs in enzymatic browning of eggplant fruit has been vastly studied and also chlorogenic acid is shown to be the most predominant phenolic in the flesh of its fruit (Whitaker and Stommel 2003; Singh et al. 2009). Studies on eggplant PPOs describing their biochemistry, enzymatic action and genes have been published (Pérez-Gilabert and García Carmona 2000; Shetty et al. 2011). But due to their role in plant defense and food quality the identification of any additional PPO genes could be critical. Therefore, the main objective of this manuscript was to identify any additional eggplant PPOs utilizing bioinformatic tools and publicly available databases.

## Materials and methods

The PPO gene sequences published by Shetty et al. (2011) were utilized to analyze public databases for additional eggplant PPOs using basic local alignment search tool (BLAST; Altschul et al. 1990) against National Center for Biotechnology Information (NCBI; http://www.ncbi.nlm.nih.blast). The *S. melangena* hits were exported to San Diego Super Computer Center biology workbench (http://seqtool.sdsc.edu) for sequence analysis. Sequences were aligned using CLUSTALW tool (Thompson et al. 1994) after translation to protein sequences. Phylogenetic relationships among the identified sequences were calculated using PHYLIP (Felsenstein 1989). Sequence identity matrix of all the thirteen PPOs was computed using LALIGN program (http://workbench.sdsc.edu/). The molecular weight (statistical analysis of protein sequence (SAPS), Brendel et al. 1992) and isoelectric point (http://seqtool.sdsc.edu/CGI/BW.cgi) of different PPO sequences were also determined. The N-glycosylation sites and their position in the mature novel PPO sequences were analyzed using NetNGlyc software (www.cbs.dtu.dk/services/NetNGlyc/). All the additional sequences obtained (except one) were almost complete with minimal gaps, therefore, were further not sequenced. All thirteen eggplant PPOs are aligned and analyzed as described above.

## Results

**Fig. 1:**
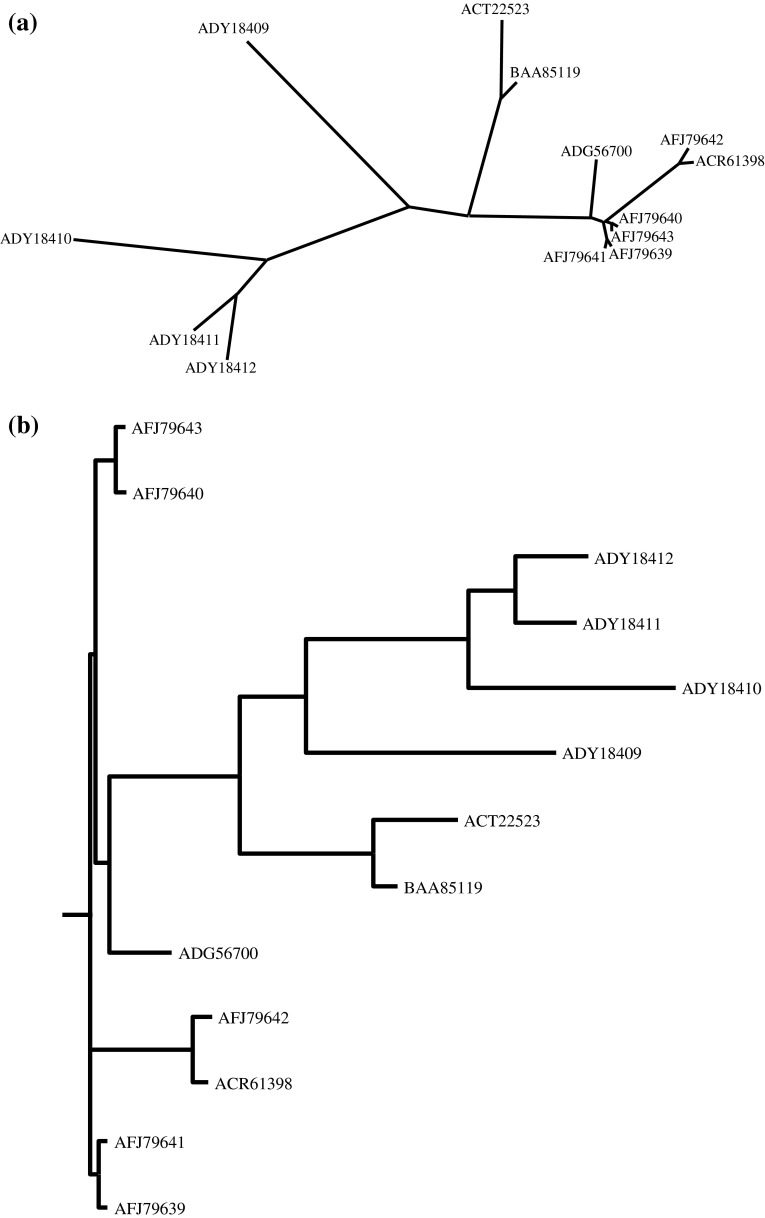
**a** Phylogenetic analysis (*unrooted tree*) of eggplant PPOs, **b** phylogenetic analysis (*rooted tree*) of different eggplant PPOs

Six PPO sequences were reported in eggplant by Shetty et al. (2011). However, large scale work on eggplant PPO ESTs/gene sequences has been reported in past few years. Therefore, a detailed analysis of PPOs in eggplant was performed; it resulted in identifying a multi-gene family of thirteen PPOs, grouped into two major clusters and a third minor group (Fig. 1). The published sequences (Shetty et al. 2011) were used as a search tool for NCBI BLAST search, seven additional eggplant PPO sequences with a sequence identity >62 % in overlaps of at least 266 amino acids were identified in this study. A phylogeny tree and evolutionary relationships of all the PPOs was constructed using PHYLIP (Fig. 1a, b). It was observed that six novel sequences identified are paired up with ADG56700 as a separate cluster and the seventh sequence (BAA85119) was closely related to ACT22523. Four previously characterized sequences (ADY184109, 18410, 18411 and 18411) form a second major cluster with ADY18409 forming a separate branch. Based on sequence comparison with the other eggplant PPOs the new sequences represent almost full length sequences except for one (BAA85119). The eggplant PPOs were compared with other plant PPOs (data not shown) including potato (*Solanum tuberosum* L.), tomato (*Lycopersicum esculentum* L.), tobacco (*Nicotiana tabacum* L.) and grape (*Vitis vinifera* L.). The tentative start site of mature PPOs is indicated based on the sequence alignment with higher plant and previously characterized eggplant PPOs. Tentative proteolytic processing sites for both stromal and thylakoid peptidases are also indicated. The length and molecular weight of mature PPOs ranged 584–601 amino acids and 65.9–67.7 kDa, respectively (Table 1). The molecular weight of mature PPOs was about ~56–58 kDa (after proteolytic processing). The isoelectric point of different PPOs was determined and it ranged from 6.062 to 7.956 (Table 1)

**Table 1:** Characteristics of eggplant PPOs

PPO sequence	Amino acid number	Molecular weight (kDa)	Isoelectric point
ACR 61398	593	66.9	6.488
AFJ 79639	594	66.5	6.062
AFJ 79640	595	66.7	6.147
AFJ 79641	594	66.6	6.147
AFJ 79642	590	66.7	6.488
AFJ 79643	595	66.7	6.236
ADG 56700	601	67.6	6.549
ADY 18409	590	67.1	7.465
ADY 18410	584	66.3	6.294
ADY 18411	586	66.0	6.162
ADY 18412	584	65.9	6.832
ACT 22523	600	67.7	7.233
BAA 85119	266	30.4	7.956

The percentage identity among the different PPOs was also calculated using LALIGN software. Eggplant PPO with GenBank accession number ACR61398 was identified to be an additional eggplant PPO. This sequence demonstrates ~61–87 % identity with the sequences used as search tool, the new sequence shows highest identity to an eggplant PPO with GenBank accession number, ADG56700 (Table 2). ACR61398 sequence has relatively higher sequence identity (71–99 %) to the additional five PPO sequences (AFJ79639, AFJ79640, AFJ79641, AFJ79642, and AFJ79643) identified in this study, being almost identical to AFJ79642 (Table 2). Another novel sequence with an accession number, AFJ79639 was similar to two other novel sequences, AFJ79640 and AFJ79643 differing only at six different amino acid (Aa) sites over a length of 595 Aa (data not shown). AFJ79639 also shows a similar trend to ACR61398 in sequence identity. AFJ79641 is ~98 % identical to AFJ79639, AFJ79640 and AFJ79643. The sequence AFJ79643 follows same trend as the other new sequences. But the amino acid identity of AFJ79642 was comparatively lower with the other novel sequences identified. Overall, the newly reported sequences vary considerably in their similarity to the search tool sequences ranging ~61–95 % (Table 2).

**Table 2:** Identity matrix of eggplant PPO sequences as percentages

	ACR 61398	AFJ 79639	AFJ 79640	AFJ 79641	AFJ 79642	AFJ 79643	ADG 56700	ADY 18409	ADY 18410	ADY 18411	ADY 18412	ACT 22523	BAA 85119
ACR 61398	100	92	92	93	99	92	87	67	61	66	66	74	71
AFJ 79639	92	100	99	98	92	99	94	71	64	70	70	75	83
AFJ 79640	92	99	100	98	92	98	93	71	64	69	69	75	82
AFJ 79641	93	98	98	100	93	98	94	70	64	70	70	75	82
AFJ 79642	99	92	92	93	100	92	89	67	61	67	67	76	71
AFJ 79643	92	99	98	98	92	100	95	70	64	69	69	75	82
ADG 56700	87	94	93	94	89	95	100	69	63	68	68	77	78
ADY 18409	67	71	71	70	67	70	69	100	65	69	68	70	75
ADY 18410	61	64	64	64	61	64	63	65	100	81	80	61	62
ADY 18411	66	70	69	70	67	69	68	69	81	100	92	66	70
ADY 18412	66	70	69	70	67	69	68	68	80	92	100	65	66
ACT 22523	74	75	75	75	76	75	77	70	61	66	65	100	93
BAA 85119	71	83	82	82	71	82	78	75	62	70	66	93	100

**Fig. 2:**
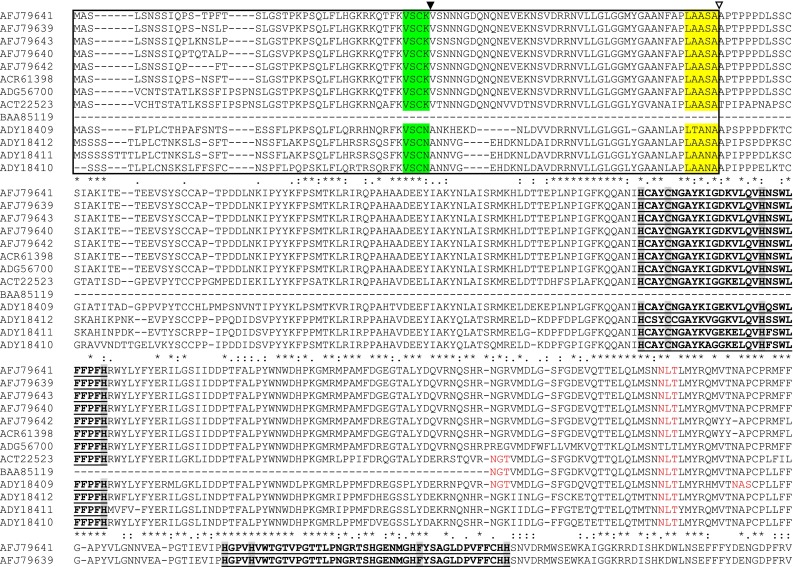

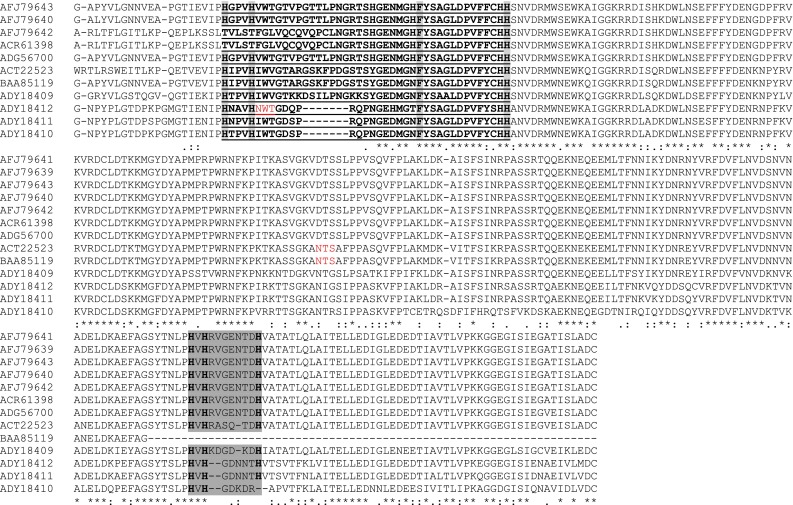
Sequence alignment of predicted amino acid sequence of thirteen eggplant PPOs. Transit peptide is boxed and proteolytic processing sites are indicated by (stromal peptidase, green) and (thylakoid peptidase, *yellow*). Conserved copper binding regions (A & B) are in bold font with underline, conserved histidine and cysteine (thio-ether linkage) are highlighted in light *grey*. Third ‘His-rich’ region is highlighted in dark *grey* at the ‘C’ terminus. N-glycosylated (Asn-Xaa-Thr/Ser) sequons are highlighted in *red*. (*) - single, fully conserved residue; (:) - conservation of strong groups; (.) - conservation of weak groups

The amino acid sequences of the newly identified PPO sequences were analyzed for determining the most conserved features of all PPOs: two copper-binding sites and transit peptide sequence (Koussevitzky et al. 1998). The chloroplast transit peptide sequence harboring both thylakoid and stromal targeting domains extending ~80–90 amino acids is also found in the novel sequences. The transit peptide sequences of all the eggplant PPOs demonstrated conserved cleavage sites for both stromal peptidases (V, S, C, K/N) and thylakoid peptidases (L, A/T, A, S/N, A; Fig. 2). Both the copper-binding (A and B) regions of all the thirteen PPOs show considerable conservation of amino acid sequence. But among the two, ‘A’ site is relatively more conserved than ‘B’. Two new sequences identified (AFJ79642 and ACR61398) in this study differed drastically from other PPOs especially in the copper ‘B’ region (Fig. 2). In these two sequences, the most conserved amino acid (histidine, ‘H’) was absent. In addition to the two known copper-binding domains of all PPOs, it was noted that the eggplant PPOs consists of a third conserved ‘Histidine (His)-rich’ region at the ‘C’ terminus (Fig. 2). NetNGlyc, N-glycosylation software identified four glycosylated sites in the different mature PPO sequences. The ‘NLT’ sequon was the most widely conserved among the different eggplant PPOs (except ADG56700). The other glycosylated sites identified are: NGT/NTS—ACT22523 and BAA85119; NGT/NAS—ADY18409.

## Discussion

During the past few years higher plant PPOs have been extensively studied due to their potential role in food biochemistry (Feillet et al. 2000) and plant defense (Constabel et al. 1995). Specifically, eggplant PPOs have been investigated due to their perceived role in browning of eggplant fruit which is rich in phenols (Shetty et al. 2011). Chlorogenic acid is the major phenolic compound present in the flesh of eggplant fruit, accounting for ~70–95 % of the total phenolics (Whitaker and Stommel 2003; Singh et al. 2009). The biological importance of PPOs demands a comprehensive study of the number and role of different PPOs present in eggplant. Analysis of publicly available data has identified seven additional eggplant PPO sequences, constituting the eggplant PPO multi-gene family of thirteen genes. The phylogenetic and identity matrix analysis of the thirteen eggplant PPOs indicate the presence of two major clusters. In addition, it was also observed that three sequences (ACT22523 and BAA85119; ADY18409) probably represent different branch/clusters, these could include a few more yet to be identified PPO sequences.

The N-terminal region of the eggplant PPOs contained chloroplast transit peptide, these regions consists of both stromal and thylakoid targeting domains. Stromal and thylakoid targeting domains help in importing the mature PPOs into chloroplast stroma and thylakoid lumen, respectively. The molecular weight of plant PPOs varies considerably probably owing to the presence of multiple genes coding for plant PPOs and also due to partial proteolysis of these enzymes. The mature plant PPO proteins are usually ~52–62 kDa (Chevalier et al. 1999). The seven novel sequences analyzed possessed the widely conserved signature motifs of all PPOs: copper A/B binding sites (Koussevitzky et al. 1998). Though the two sites were mostly conserved there were some noticeable amino acid substitutions and deletions especially in copper ‘B’ region. Further, two PPOs in particular (AFJ79642 and ACR61398) exhibited considerable deviation in the conserved copper ‘B’ region compared with other sequences. This could indicate the difference in structural classes or function of different PPOs in eggplant. In addition, the presence of a third ‘His-rich’ region has been reported in potato (Hunt et al. 1993), tomato (Shahar et al. 1992) and pokeweed (Joy et al. 1995). However, the biological significance of the third ‘His-rich’ in the plant species is unknown.

The previously characterized eggplant PPOs (Shetty et al. 2011) were present in several tissues including root, leaves (young and mature), flowers (pre- and post-anthesis) and fruit. Considering the tissue from which these sequences have been reported, it appears that two genes, ACR61398 and BAA81159 are expressed in fruit. Further, based on sequence comparison and identity matrix (Table 2) data we could speculate the expression of the other novel PPOs identified in this study. Even though ACR61398 (from fruit) and AFJ79642 are highly identical, AFJ79642 is not expressed in mature fruit tissue (data not shown). This is surprising especially in view of its close proximity to ACR61398, but AFJ79642 could still be expressed at early stages of fruit development. Semi-quantitative PCR data has shown high levels of expression of ADG56700 in root and young leaves, with reduced expression levels in pre-anthesis flowers and fruit (Shetty et al. 2011). Therefore, the remaining four eggplant PPOs (AFJ79639, AFJ79640, AFJ79641 and AFJ79643) for which the tissue specificity is unknown, it is possible that these might be expressed in any of the following tissues: root, young leaves, pre-anthesis flowers and fruits. Data reported in this manuscript presents a systematic characterization of several additional eggplant PPOs. Based on sequence information some of these additional eggplant PPOs identified could be expressed in fruit. This information will aid in identifying different PPO genes that are primarily involved in browning of eggplant fruit. In addition, the data presented in this manuscript could help in better understanding the implicated role of eggplant PPOs in defense against pests and pathogens.
